# White matter regeneration induced by aligned fibrin nanofiber hydrogel contributes to motor functional recovery in canine T12 spinal cord injury

**DOI:** 10.1093/rb/rbab069

**Published:** 2021-11-29

**Authors:** Zheng Cao, Weitao Man, Yuhui Xiong, Yi Guo, Shuhui Yang, Dongkang Liu, He Zhao, Yongdong Yang, Shenglian Yao, Chuzhong Li, Lingyun Zhao, Xiaodan Sun, Hua Guo, Guihuai Wang, Xiumei Wang

**Affiliations:** 1 State Key Laboratory of New Ceramics and Fine Processing, Key Laboratory of Advanced Materials, School of Materials Science and Engineering, Tsinghua University, Beijing 100084, China; 2 Department of Neurosurgery, Beijing Tsinghua Changgung Hospital, School of Clinical Medicine, Tsinghua University, Beijing 102218, China; 3 Center for Biomedical Imaging Research, Tsinghua University, Beijing 100084, China; 4 Department of Orthopedics, Dongzhimen Hospital, Beijing 100007, China; 5 School of Materials Science and Engineering, University of Science and Technology Beijing, Beijing 100083, China; 6 Beijing Neurosurgical Institute, Beijing Tiantan Hospital, Beijing 100070, China

**Keywords:** aligned fibrin hydrogel, spinal cord injury, white matter regeneration, diffusion tensor imaging, canine

## Abstract

A hierarchically aligned fibrin hydrogel (AFG) that possesses soft stiffness and aligned nanofiber structure has been successfully proven to facilitate neuroregeneration *in vitro* and *in vivo*. However, its potential in promoting nerve regeneration in large animal models that is critical for clinical translation has not been sufficiently specified. Here, the effects of AFG on directing neuroregeneration in canine hemisected T12 spinal cord injuries were explored. Histologically obvious white matter regeneration consisting of a large area of consecutive, compact and aligned nerve fibers is induced by AFG, leading to a significant motor functional restoration. The canines with AFG implantation start to stand well with their defective legs from 3 to 4 weeks postoperatively and even effortlessly climb the steps from 7 to 8 weeks. Moreover, high-resolution multi-shot diffusion tensor imaging illustrates the spatiotemporal dynamics of nerve regeneration rapidly crossing the lesion within 4 weeks in the AFG group. Our findings indicate that AFG could be a potential therapeutic vehicle for spinal cord injury by inducing rapid white matter regeneration and restoring locomotion, pointing out its promising prospect in clinic practice.

## Introduction

Spinal cord injury (SCI) is one of the most common and severe neurological diseases that lead to variable degrees of sensorimotor deficit, sphincter disturbances and multi-system complications [1]. With over 760 000 newly diagnosed cases annually worldwide and a high disability rate, SCI has been a great challenge for public health services [2]. Although an increasing number of neuroprotective and neuroregenerative pharmaceuticals (e.g. naloxone, riluzole, anti-Nogo) have been introduced to facilitate spinal cord repair, few have achieved satisfactory results in clinical practice [3]. Neuromodulatory interventions, including spinal cord stimulation and brain stimulation, rely on the intact circuit in the spared spinal cord, resulting in limited effects on severe SCI [4]. Moreover, diverse cell-based therapies remain to be assessed due to ethical and tumorigenesis concerns [3]. An important barrier to improve spinal cord regeneration with the abovementioned therapies is that the altered microenvironment at the injured site cannot support nerve tissue regrowth. Inappropriate extracellular matrix (ECM) with increased extrinsic inhibitors (e.g. myelin-associated proteins), decreased growth factors and abnormal micro-architecture limit the self-healing capacity of the spinal cord post injury [5–7]. Therefore, a possible approach to overcome these obstacles is to deliver multiple bioactive agents *in situ* through various delivery systems (e.g. tissue-engineered bio-scaffolds) to regulate the considerably altered microenvironment. It is noteworthy that most existing studies focus on the optimization of the combination pattern of bio-activated agents. Limited studies have made great efforts to enhance the repair efficacy by improving the bio-scaffold itself, especially by simultaneously modifying the structures and mechanical properties of the scaffold to augment its biophysical advantages. Moreover, bio-scaffolds with simplified components will be easily applied into clinical practice due to minimized biosafety concerns. Therefore, it needs to be further clarified whether it is feasible to improve the outcome of spinal cord repair by optimizing the bio-scaffold itself without the combination of extra pharmaceuticals and cells.

The white matter in the spinal cord mainly consists of ascending tracts (e.g. fasciculus gracilis, fasciculus cuneatus, spinothalamic tract) and descending tracts (e.g. corticospinal tract, rubrospinal tract, vestibulospinal tract), which are the anatomical bases of sensor and motor functions, respectively [8]. Functional restoration after SCI is based on the reconnection and remyelination of axons in impaired tracts, indicating the critical role of white matter regeneration in the functional reconstruction of the injured spinal cord [9]. Additionally, adjuvant neuromodulatory intervention and rehabilitation therapy during the long-term recovery of SCI cannot work without sufficient regenerated axonal fibers to conduct signals in the white matter tracts, emphasizing the importance of white matter regeneration for the whole course of SCI treatments [10, 11]. Of note, impaired white matter has the potential to regrow without any exogenous cells if the corresponding neuronal cell bodies have not been destroyed, even with limited capacity [12, 13]. However, in severe SCI, the cavities formed in the lesion core and surrounding scar formed by either astrocytes or stromal cells prevent the endogenous axonal regrowth in the white matter, highlighting the importance of ECM alteration in promoting white matter repair [14]. Diverse bio-scaffolds were introduced to bridge the gap between the rostral and caudal stumps of the injured spinal cord [15–19]. However, few bio-scaffolds mimic the aligned structure of the white matter at the micro- and nanoscale and the softness of the ECM at the same time. In our previous study, a hierarchically aligned fibrin nanofiber hydrogel (AFG) was designed and fabricated, which can act as an extraneous orientated cable with a bionic structure of white matter bundles to support tissue regrowth. The effect of AFG on stem cell differentiation into neurocyte *in vitro* has been clarified, demonstrating its appropriate mechanical and structural features for nerve cell survival [20]. Furthermore, it has been proven that *in situ* AFG implantation accelerates axonal regrowth and myelination in rats with SCI, implying the possibility and feasibility of promoting SCI repair using AFG in large animal models [21]. Because the pathophysiological processes after SCI in rodents exhibit considerable differences from those in primates, especially for long-term functional restorations [22, 23], we further evaluated AFG using a canine L2 spinal cord hemisected defect model, showing positive effects on inducing axonal regeneration and motor functional recovery [24]. Nevertheless, we need to consider that variations in regenerative potentials and cellular responses exist between different animal species and even different damaged segments. Instead, the canine thoracic segment SCI model can better simulate the dynamic and clinical conditions in human patients with SCI but has not yet been used to evaluate the efficacy of AFG.

In this study, the AFG was applied to a beagle canine with hemisected T12 SCI to evaluate its ability to facilitate white matter regeneration after SCI. During the 16 weeks of observation after injury, the processes of white matter regeneration were dynamically traced using a novel high-resolution magnetic resonance imaging (MRI) methodology, that is, optimal multi-shot diffusion tensor imaging (DTI) sequence, which has not been previously reported. The hindlimb motor function was observed and assessed with the Olby’s scores in both static and movement conditions. Moreover, detailed histological analysis was performed to evaluate white matter regeneration with the involvement of AFG. Briefly, we provided sufficient preclinical evidence for the potential of AFG implantation for SCI treatment. Furthermore, we clarified the evaluation of the novel DTI methodology in clinical assessment of patients with SCI.

## Materials and methods

### Hydrogel fabrication

AFG was prepared by electrospinning with a self-assembled solution collection process [20]. Fibrinogen (Sigma Aldrich, St. Louis, MO, USA) was dissolved in ultra-pure water and mixed with polyethylene oxide (PEO, Sigma Aldrich) at 8 mg/ml. The precursor solution was injected into the collection container at a speed of 2 ml/min with a voltage of 4.5 kV (Spellman, Hauppauge, NY). The collection container was connected to the ground and filled with 50 mM CaCl_2_ and 5 U/ml thrombin (Sigma Aldrich) solution. The fibrin fibers were formed and stretched with the container rotated at 60 rpm, when the fibrinogen precursor contacted the collection solution and then polymerized to fibrin. Accumulated fibrin fibers were collected as fibrin hydrogel bundles with adjustable diameters.

### Characterization of hydrogel

To characterize the microstructure of the AFG and AFG/fSAP hydrogels, the samples were fixed with 2.5% glutaraldehyde at 4°C overnight and then dehydrated through graded concentrations of ethanol (30%, 50%, 70%, 80%, 90%, 95% and 100%, w/v; 15 min × twice for each grade). Finally, the samples were critical-point dried with CO_2_ and were imaged with a field-emission scanning electron microscope (SEM; JEOL 6700F; Tokyo, Japan). For transmission electron microscope (TEM) exanimation, after fixation with 2.5% glutaraldehyde, the samples were further fixed with 1% osmic acid for 1.5 h. Then the samples were dehydrated through graded ethanol and embedded in Epoxy Embedding Medium (Sigma Aldrich, MO, USA) overnight, followed by polymerization at 60°C for 10 h. Seventy nanometer-thick axial ultrathin sections were cut with an ultramicrotome (EM UC6, Leica, Germany) and stained with uranyl acetate and lead citrate. Images were obtained with a TEM (Hitachi H-7650B; Hitachi, Tokyo, Japan).

The stiffness of the hydrogels was measured with an atomic force microscope (AFM, Dimension ICON, Bruker, Billerica, MA, USA). AFM cantilever tips of V-shaped silicon SNLD probes (nominal spring constant 0.06 N/m) were modified by attaching 20-μm diameter glass beads. The measurement was conducted at 500 points randomly on each hydrogel. The Young’s moduli of all hydrogels were determined by fitting the force-indentation plots with the Hertz model.

### Animal surgery and AFG transplantation

Canine surgeries were performed in an animal laboratory in Beijing Tiantan Hospital. The procedures were approved by the Institutional Animal Care and Use Committee of the Beijing Neurosurgical Institute (No. SYXK(Jing)2013-0009). A total of 12 adult female Beagle dogs (weighing 10 ± 1 kg, Fang Yuanyuan Inc., Beijing, China) were normally fed in a single cage per dog in a constant temperature and humidity environment.

**Figure 1.**
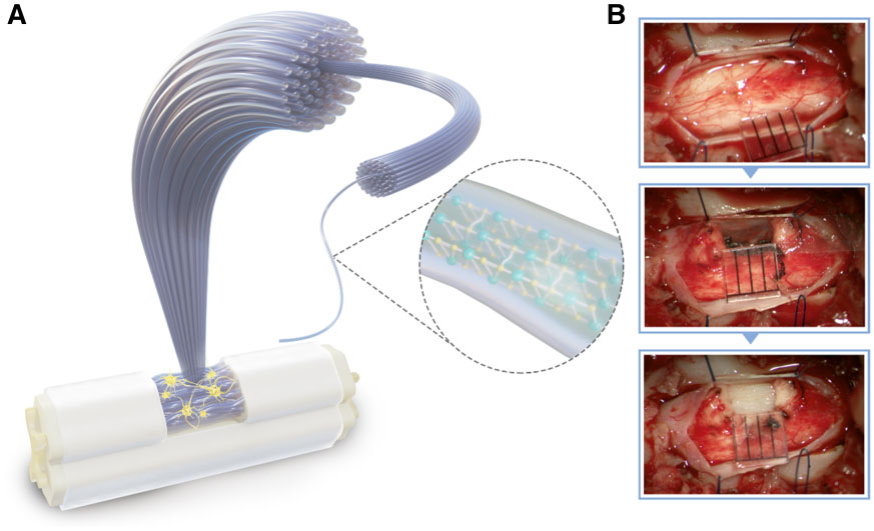
Surgery process of canine traumatic hemisection T12 SCI model. (**A**) Diagram of AFG and (**B**) the SCI surgery and AFG transplantation process.

All dogs underwent fasting for 12 h before being anesthetized with pentobarbital sodium (30 mg/kg). Laminectomy was performed to expose the T12 spinal segment, and the endorhachis incision was carefully performed under a microscope (OPMI, Zeiss, Oberkochen, Germany). A 4-mm right-side portion of the spinal cord was removed using microscissors after the dorsal vascular closure at 4–5 mm in length with electrocoagulation. The spinal cord gap was obtained under the exposed endorhachis with a sharp force (Fig.  1B and Supplementary Video S1). To prevent hemorrhage and injury to the left portion of the spinal cord, the spinal dorsal vessel at the right side was first interdicted by bipolar coagulation (Supplementary Fig. S1A). The original incision located at the posterior median fissure passed through the midcourt line to the anteromedian groove and avoided the spinal ventral vessel (Supplementary Fig. S1B and D and Video S1). In the AFG group, the AFG hydrogel bundle was sterilized in 75% ethanol, washed in 0.9% saline preoperatively, cut into appropriate size, and transplanted to fill the gap (Fig.  1B). In the control group, after the hemisection, no any materials but only saline was used to fill the gap. The endorhachis incision was sutured and covered with an artificial dura mater. Finally, the wound was sewn and banded. Each dog routinely received cephalosporins (0.5 g, intravenous, once per day) during surgery.

Postoperatively, the dogs were fed with ibuprofen in food for 3 days and received antibiotics subcutaneously for 5 days. Due to the lack of normal urination, manual massage was performed twice per day to assist in micturition until bladder function recovery. Moreover, we massaged the hindlimb muscles and leg joints of the dogs to prevent severe atrophy. When the dogs recovered and the dorsal wound healed, the dogs walked daily for at least 1 h.

### Somatosensory evoked potential

Somatosensory evoked potential (SEP) measurements were performed with a needle electrode and electrodiagnostic system equipped with Medelec Synergy software (Natus Neurology, Pleasanton, CA, USA). Dogs were intravenously anesthetized with pentobarbital sodium (30 mg/kg) and kept lying flatly with vertical and shank easy-to-imbed electrodes. Positive and negative electrodes were inserted near the ankle subcutaneously to stimulate the posterior tibial nerve, which could explicitly observe the dog’ s paw tremble slightly, and the recording electrode was located at the skull surface of the sensory area of the cerebral cortex. Signals of the two legs were captured at two time points, before and after the lesion was completed. The grounding electrode was inserted into the foreleg.

### DTI analysis

DTI datasets were obtained by MRI with a Philips Achieva 3.0 T TX system, using multi-shot echo planar imaging DTI sequence [25, 26]. The dogs were anesthetized as before and lying upturned in a stereotypic posture with the spine stretched straight. The DTI sequence was operated with the parameters shown below. The scan orientation was transverse, the field of view (FOV) was 130 × 79 × 63, displayed as RL × AP × FH (RL, right-left; AP, anterior-posterior; FH, foot-head), the acquisition matrix was 132 × 76 × 21, the resolution was 1 × 1 × 3 mm^3^, number of signal averages was 2, number of shots was 4, TR/TE = 4001/54/93 ms, *b*-value = 800 s/mm^2^. The DTI data were gathered postoperatively for 1 day, 1, 4, 8 and 16 weeks, respectively, for each dog. Image reconstruction was performed according to a previously published method [26]. All reconstruction procedures were implemented using MATLAB. Fiber orientation estimation was performed in the MRtrix framework using constrained spherical deconvolution [27]. FA values were calculated using DTI studio software in integrated, hemi-excised portions and the corresponding contralateral ROI of the impaired spinal cord DTI transverse slices.

### Behavioral assessment

Olby scores were used to measure the motor function of canine hindlimbs after SCI surgery [22]. Dogs freely walked in an open area for at least 1 h at 1 day and 1, 4, 8 and 16 weeks after SCI surgery. The activity of the hindlimbs, and joints and weight-bearing condition of the right hindlimbs were evaluated according to the Olby scoring scale. The mean scores were calculated in a double-blind study.

### Histological analysis

Spinal cord tissues were harvested 16 weeks after AFG transplantation surgery. The dogs were anesthetized and perfused with 0.9% saline and cold 4% formaldehyde through the heart to immobilize the tissue. The spinal cord from T11–T13 was retrieved and fixed in 4% formaldehyde for another 48 h.

The harvested tissues were cryopreserved in the optimal cutting temperature compound, and the tissue sections were sliced longitudinally into 10-μm thickness using a freezing microtome (CM1900, Leica Microsystems, Germany). Sections were stained with H&E to survey the lesion and adjacent tissue with cellular and ECM. Images were obtained using a Zeiss Axio Scan Z1 slide scanner (Carl Zeiss Microscopy, Germany), and representative images were processed using ZenBlue software. For each sample, photographs were obtained from random fields of ROI during light microscopy observation. The images were quantitatively analyzed using ImageJ software (Softonic, Barcelona, Spain).

### Immunofluorescence

For immunofluorescence staining, the sections were blocked in 10% goat serum and 0.3% Triton-100 to prevent non-specific binding and improve permeability. The sections were incubated with primary antibodies (rabbit anti-tubulin-βIII, 1:800; Abcam, Cambridge, UK), mouse anti-glial fibrillary acidic protein (GFAP, 1:300, Abcam), mouse anti-neurofilament (NF, 1:800, Abcam), rabbit anti-growth associated protein-43 (GAP43, 1:500, Abcam), and rabbit anti-tyrosine hydroxylase (TH, 1:500, Abcam) at 4°C overnight. Then, the sections were washed three times with phosphate buffered saline and incubated in secondary antibodies (anti-rabbit-594, 1:800, Invitrogen, Waltham, MA, USA) and anti-mouse-488 (1:800, Invitrogen) for 1 h at room temperature in the dark. Finally, the secondary antibodies were washed and sections were mounted in medium containing 4′,6-diamidino-2-phenylindole (DAPI, Abcam). Each slide was scanned by laser confocal fluorescence microscopy (LSM s780; Carl Zeiss Meditec AG, Germany), and the images were processed using ZenBlue software.

### Statistical analysis

Quantitative analysis of IF images was performed using ImageJ software (version 1.52a Wayne Rasband National Institutes of Health, USA, https://imageJ.nih.gov/ij). For measuring each parameter (area or cell counts with positive expression), 10 ROIs were randomly selected in different lesion portions. Values are presented as mean ±standard deviation (SD). Statistical analyses were performed using SPSS (version 25.0, IBM Corp., New York, USA) with a two-tailed unpaired Student’s *t* test. *P* values < 0.05 were considered statistically significant.

## Results

### AFG fabrication and characterization

AFG fabricated via electrospinning and collected using a rotated water bath containing thrombin and calcium ions exhibited good flexibility and ductility with an easily adjustable diameter and length (Supplementary Fig. S2A and B). The SEM images of the longitudinal sections of the AFG showed an aligned structure of nanofibers, which is similar with the structure of ECM in white matter of spinal cord (Supplementary Fig. S2C–E). The Young’s moduli of AFG measured by AFM were 3.08 ± 0.28 kPa.

### Host tissue ingrowth with assistance of AFG

For canine hemisected SCI, several AFG short sticks of approximately 1 mm in diameter and 4 mm in length were stacked in parallel in the gaps (Fig.  1 and Supplementary Video S1). To guarantee hemi-excision of the spinal cord, SEP was monitored during surgery (Supplementary Fig. S3), which showed a dramatically attenuated signal of the right hindlimb after right hemisection of the spinal cord, in contrast to the stable SEP signal of the left hindlimb pre- and postoperatively.

**Figure 2.**
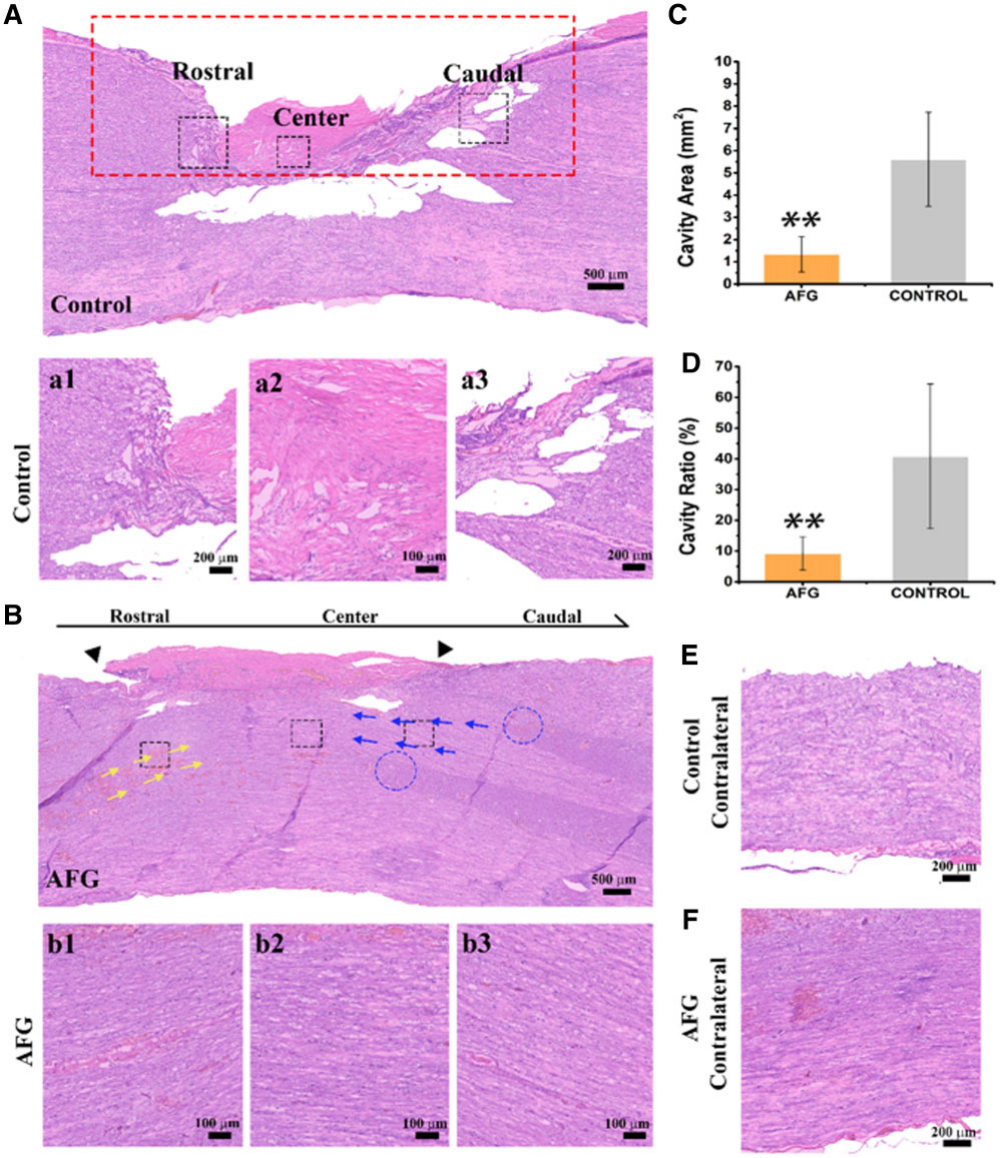
H&E Staining of the global and regional view of the spinal cord lesion. (**A**) Holistic H&E stain image of control spinal cord and (**a1**), (**a2**), and (**a3**) refer to the black squares in rostral, center, and caudal region of control spinal cord, respectively. (**B**) Holistic H&E stain image of AFG spinal cord, triangles indicated the lesion range, yellow and blue arrows marked the tissue orientation, and blue circles signed the broken ends of white matter and gray matter. Black squares refer to the (**b1**) rostral, (**b2**) center, and (**b3**) caudal of AFG spinal cord from the right to left, respectively. (**C**) Calculated values of cavity area in AFG and control spinal cord. (**D**) Cavity ratio compared between AFG and control spinal cord. (**E**) Contralateral spinal cord of the control group in the Central region. (**F**) Contralateral spinal cord of the AFG group in the Central region (*n* = 3, **P < 0.01).

Spontaneous recovery following SCI generally leads to constant degeneration of the ECM and forms disordered tissue structure [28]. At 16 weeks after SCI, the hematoxylin-eosin (H&E) staining images showed cavities and scar tissues with randomly infiltrated host cells and disordered deposited ECM in the lesion site of the control group, which blocked the reconnection between the rostral and caudal stumps of the spinal cord via the regenerated nerve fibers (Fig.  2A). In contrast, histologically obvious white matter composed of a large area of consecutive, compact, and aligned nerve fibers regenerated in the lesion area with assistance of implanted AFG, which had no significant morphological difference and obvious interface with surrounding native tissues (Fig.  2B). Nevertheless, it is noted that SCI leads to damage to both white matter and gray matter. The clear-cutting ends of gray matter in the lesion site (marked in blue dotted circles) were still visible at 16 weeks postoperatively, indicating their limited regenerative capacity because of loss of neurons. Although the AFG did not dramatically promote gray matter regeneration, it did significantly induce good white matter regrowth, its effect on functional recovery of the spinal cord was worthy of further evaluation. Moreover, AFG could help ameliorate the progressive deterioration of the contralateral spinal cord, unlike the distinctly atrophied spinal cord in the control group (Fig.  2E and F). The total cavity area in the AFG group (1.34 ± 0.79 mm^2^) was significantly smaller than that in the control group (5.60 ± 2.12 mm^2^, *P* < 0.05) (Fig.  2C). Furthermore, the cavity ratio (ratio of cavity area to hemisected area) of the AFG group (9% ± 5%) was significantly lower than that in the control group (40% ± 23%, *P* < 0.05) (Fig.  2D). These results suggest that AFG implantation could promote infiltration of host nerve tissue into the lesion site, reconnection of the rostral and caudal injured matters, and eventually reduction in cavity formation.

In addition, 16 weeks after the implantation, the AFG has been completely degraded since we did not observe any hydrogel left in the lesion site in the H&E staining images. That is consistent with our previous studies that the majority of the AFG could be degraded and absorbed around 1 week *in vivo* [24].

### White matter regrowth identified with DTI

**Figure 3.**
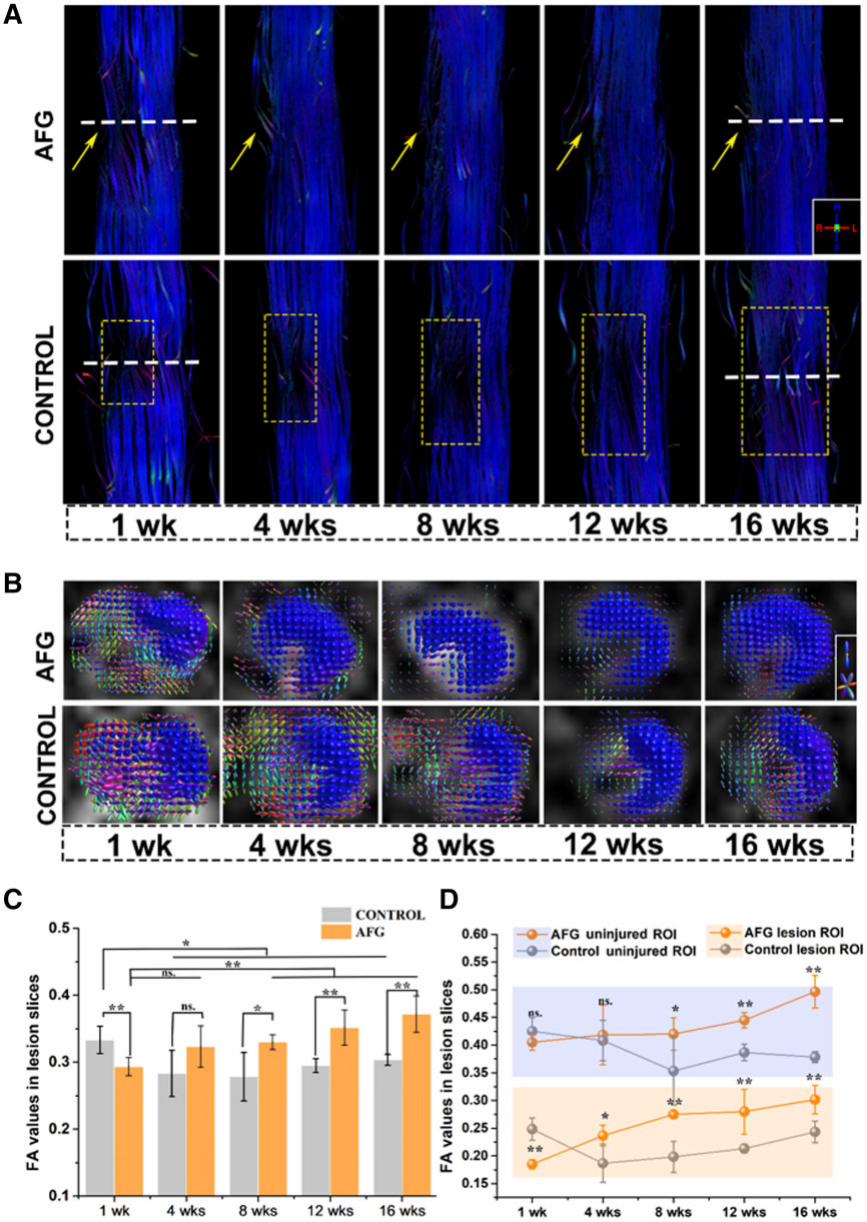
Sequential directional DTI tractography and FA statistical values. (**A**) Sequential directional DTI fiber track of AFG spinal cord and control spinal cord. Yellow arrows and squares highlight the lesion gap, and the dotted lines point out the epicenters. (**B**) Directional traverse DTI views of AFG spinal cord lesion epicenter from 1 to 16 weeks, and the traverse views of control lesion epicenter from 1 to 16 weeks. (**C**) Average FA values comparison between AFG and control spinal cord in center lesion slices. (**D**) Respective FA values and tendency in AFG uninjured regions (orange line in blue shadow) and lesion regions (orange line in orange shadow), control uninjured regions (gray line in blue shadow), and lesion regions (grey line in blue shadow). (*n* = 3, *P < 0.05, **P < 0.01, ns.: no significant difference).

MRI is the best non-invasive methodology to visualize the microstructure and orientation of the spinal cord *in vivo*. Particularly, DTI tractography has unique advantages in qualitatively analyzing highly anisotropic nerve fibers and spinal cord white matter integrity [12]. Here, we developed a new scanning sequence of DTI and traced the regenerative nerve fibers antegradely and retrogradely in the injured sites of spinal cords with or without AFG. DTI was used to perform a follow-up assessment of the canine spinal cords at intervals of 1, 4, 8, 12 and 16 weeks after SCI. In DTI tractography images, the blue color denotes diffusion of water in the direction of the static magnetic field, which is nearly parallel to the long axis of the spinal cord, therefore representing the aligned nerve fibers with high anisotropy. Other colors indicate the distortions of water diffusion due to injury. At 1 week postoperatively, DTI tractography images showed obvious defects around the epicenter of the lesions in all injured spinal cords, confirming complete disruption of the sectioned nerve fiber bundles in both the control and AFG groups postoperatively (Fig.  3A). After 4 weeks, there were a few fiber bundles that were successfully tracked across the spinal cord defect in the AFG group. Moreover, anisotropy in the hemisected sites increased gradually over time, indicating that the AFG accelerated the white matter regrowth with newborn nerve fibers aligned along the rostrocaudal direction and retained the integrity of white matter in the spinal cord. In contrast, the defects in the control group were still detectable after 16 weeks because of the progressive deterioration of lesion tissues, with gradual expansion toward the rostral and caudal ends.

Furthermore, DTI in the transverse view from the epicenter of SCI provided more intuitionistic images to visualize the regeneration of spinal cord white matter over time, as shown in Fig.  3B. The circular region of interest (ROI) in both groups at week 1 confirmed the hemisection of the spinal cords (Fig.  3B). Hereafter, the area of the blue signals increased gradually over time in the AFG group, which was associated with the aligned nerve fibers regrown in the defect site. However, the area of the ROIs did not increase in the control group during the entire observation period but slightly decreased instead. Fractional anisotropy (FA) is a frequently used DTI parameter that quantitatively depicts the anisotropy of diffusion, with a higher value (closer to 1) of anisotropic normal tissues and lower value (closer to 0) of isotropic lesion sites. The average FA value calculated from the integral ROI of the spinal cords in the AFG group gradually increased over time from the first week after SCI, while, in the control group, it showed a decreasing tendency until the fourth week and remained at a low level in the following time points (Fig.  3C). Furthermore, the FA values from the injured half and uninjured half of the spinal cord were calculated, which exhibited decreased FA values at the lesion regions in both the AFG and control groups (Fig.  3D). Moreover, the FA values in the AFG group were significantly higher than those in the control group, regardless of the injured or uninjured side, which implied that the AFG assisted the axonal regeneration of the injured spinal cord and diminished the adverse effects on the contralateral tissue. Furthermore, it is noted that the FA values from the uninjured side in the control group continued to decline until 8 weeks after SCI, indicating the enlargement of the lesion that might be induced by the secondary injury (Fig.  3D and Supplementary Fig. S4).

### Locomotor behavior recovery

**Figure 4.**
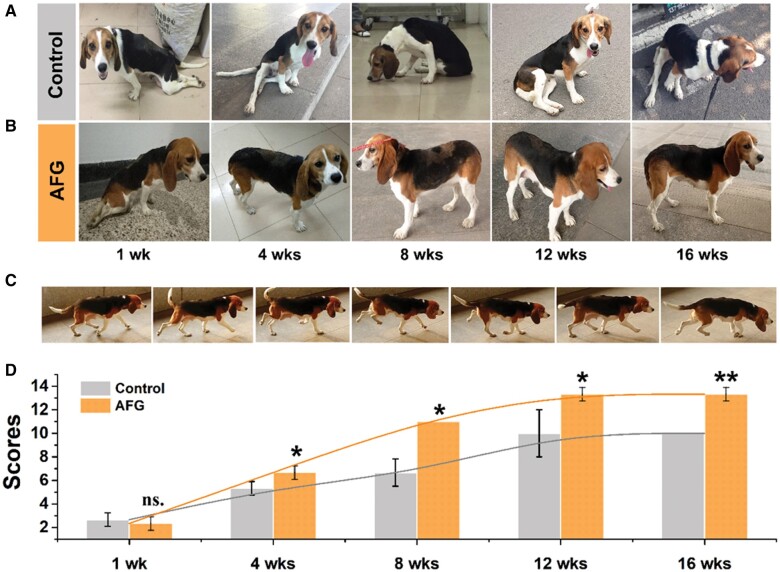
Static standing position and movement behavior evaluation. (**A**) Postures of control dogs and (**B**) AFG dogs from 1 week to 16 weeks postoperatively. (**C**) Walking decomposition movements of AFG canine at 12 weeks. (**D**) Obly scores of the dogs in AFG and control groups from 1 week to 16 weeks. (*n* = 3, *P < 0.05, **P < 0.01, ns.: no significant difference).

Behavior analysis is crucial in evaluating the functional restoration of the spinal cord after SCI. Dynamic changes in static standing position and movement behavior were observed throughout 16 weeks postoperatively (Fig.  4). In the first 2 weeks, the dogs had hindlimb limp and feeble (Fig.  4A and B) and then gradual improvement of locomotion. At 3–4 weeks postoperatively, the dogs in the AFG group showed fragility with the right legs slightly bearing their weights. The static standing position and weight bearing of the dogs in the AFG group were almost normal at 7–8 weeks postoperatively (Fig.  4B). After 12 weeks, the right legs could bear the whole body weight well, allowing dogs to keep in the climbing position with good stability for a while (Supplementary Fig. S5) with their center of gravity on the hindlimbs. Moreover, the dogs in the AFG group could walk with continuous and robust support of the hind legs, especially the right legs (Fig.  4C). However, dogs in the control group were still incapable of controlling their right legs and maintaining good balance in standing and walking, although the muscle strength of the right leg had some recovery.

Obly scores were used to evaluate locomotor performance and recovery status after SCI [22]. All dogs presented progressive recovery of locomotion after SCI surgery with increasing Obly scores. The dogs in the AFG group exhibited significantly higher Obly scores than those in the control group at the fourth week postoperatively (Fig.  4D). In the AFG group, the average Obly score increased from 2.3 ± 0.6 at week 1–13.3 ± 0.6 at the 12th week with nearly normal gait, while the highest score of the control group was approximately 10. The locomotor performances of the dogs were recorded, as shown in Supplementary Videos S2 and S3, which revealed that dogs with AFG implantation had much better gait and coordination compared to the control dogs. Moreover, it is noteworthy that, at the 8th week postoperatively, the dog in the AFG group could climb the step easily, however, the dog in the control group had a problem completing the action (Supplementary Video S4). These results indicated that AFG implantation facilitated functional restoration during the recovery of SCI, in accordance with the histological and imaging results mentioned above.

### Regeneration of damaged white matter tracts confirmed by immunofluorescence

**Figure 5.**
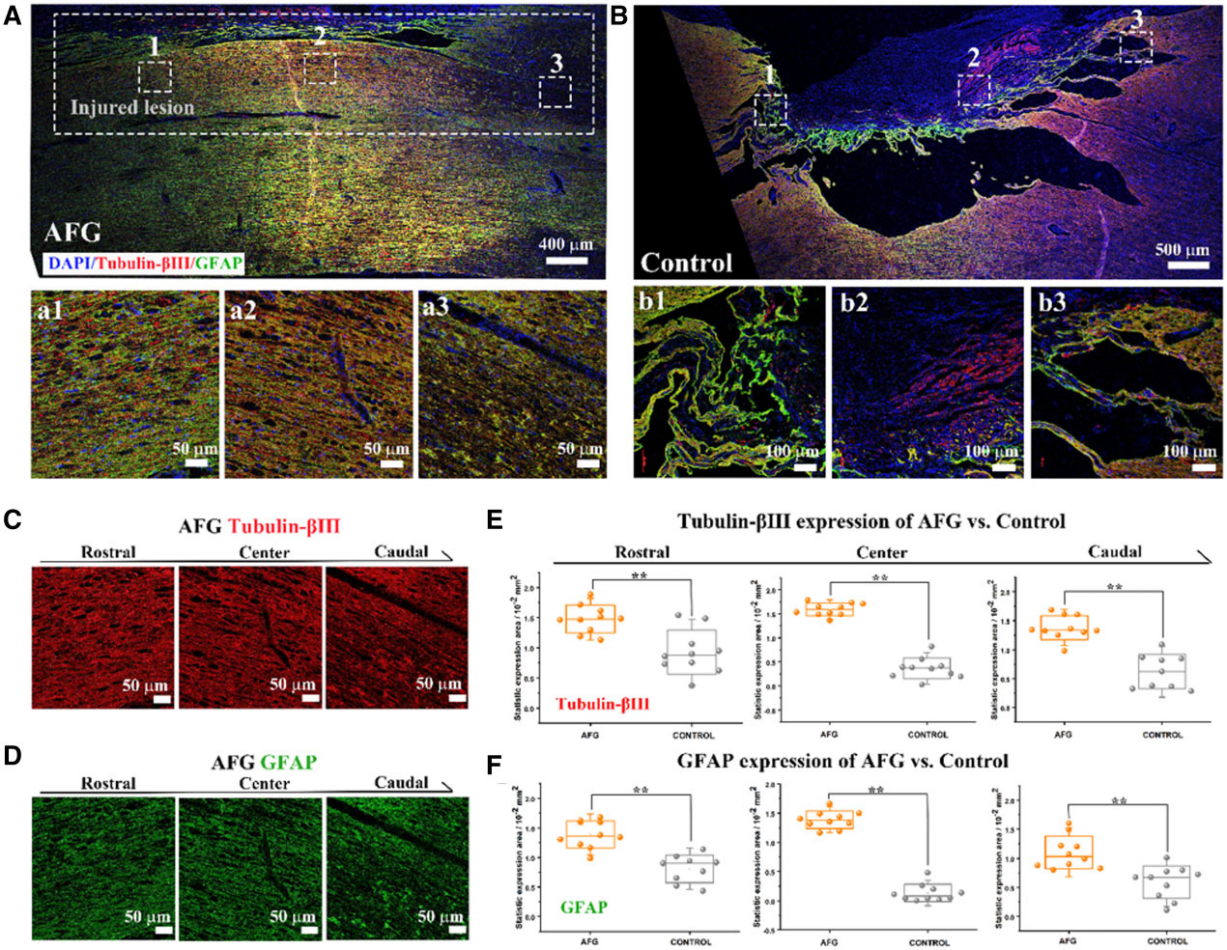
Immunofluorescence stain and analysis of spinal cord in the AFG and control groups. (**A**) Full view of the hemisected injured spinal cord of AFG with tubulin-βIII^+^ in red, GFAP^+^ in green, and DAPI in blue, and a1–a3 showed the rostral, center and caudal portion, with respect to the three squares in full view marked with number. (**B**) Immunofluorescence full view of the spinal cord in the control group and b1–b3 showed enlarged images of the numbered squares in B respective to rostral, center, and caudal part. (**C**) Tubulin-βIII^+^ staining images of AFG in rostral, center, and caudal regions. (**D**) GFAP^+^ staining images of AFG in rostral, center, and caudal regions. (**E**) Statistical analysis of the immunofluorescence expression in tubulin-βIII compared between AFG and control groups in rostral, center, and caudal lesion part. (**F**) Expression comparison of GFAP between AFG and control in rostral, center, and caudal lesion part. (**P < 0.01).

Regeneration of damaged white matter tracts that includes ingrowth of neuronal populations, axonal regrowth and myelination, increase in white matter integrity, and reconnection of ascending and descending nerve pathways plays critical roles in functional restoration after SCI. To further confirm the regenerated white matter tracts that may correlate with the locomotor outcomes, immunofluorescence-stained tissue sections were examined using tubulin-βIII (red) and GFAP (green) as markers for neurons and astrocytes, respectively (Fig.  5). In the AFG group, the majority of the regenerated tissue expressed tubulin-βIII and GFAP (Fig.  5A), regardless of the rostral, caudal, or center areas (Fig.  5a1–a3, C and D), which had no evident differences with the contralateral intact spinal cord, indicating that the regenerated tissues shown in histological H&E staining and DTI tractography images dominate neural tissues. However, in the epicenter of the lesion in the control group, most regrowth tissues did not express tubulin-βIII or GFAP (Fig.  5B and Supplementary Fig. S4A–D). Although the regrowth tissues near the rostral and caudal ends had a low tubulin-βIII and GFAP positive expression, they were arranged in disordered and discrete pattern (Fig.  4b1–b3). Quantitative analysis showed that the areal density of tubulin-βIII^+^ and GFAP^+^ tissue from rostral, epicenter, or caudal regions in the AFG group was significantly higher than that in the control group (Fig.  5E and F). Particularly, in the epicenter of the lesion, the spinal cord of the AFG group exhibited obviously higher expression activity (ratio of fluorescence intensity to cell counts) in tubulin-βIII and GFAP (Supplementary Fig. S6E), which should be attributed to the densely packed axons regenerated with the assistance of the AFG bridge. We also noted that some non-nervous tissue inevitably grew edgeways because of the existence of the damage endorhachis (Supplementary Fig. S7A and B), which showed DAPI-positive staining and tubulin-βIII/GFAP-negative staining (Supplementary Fig. S7C).

**Figure 6.**
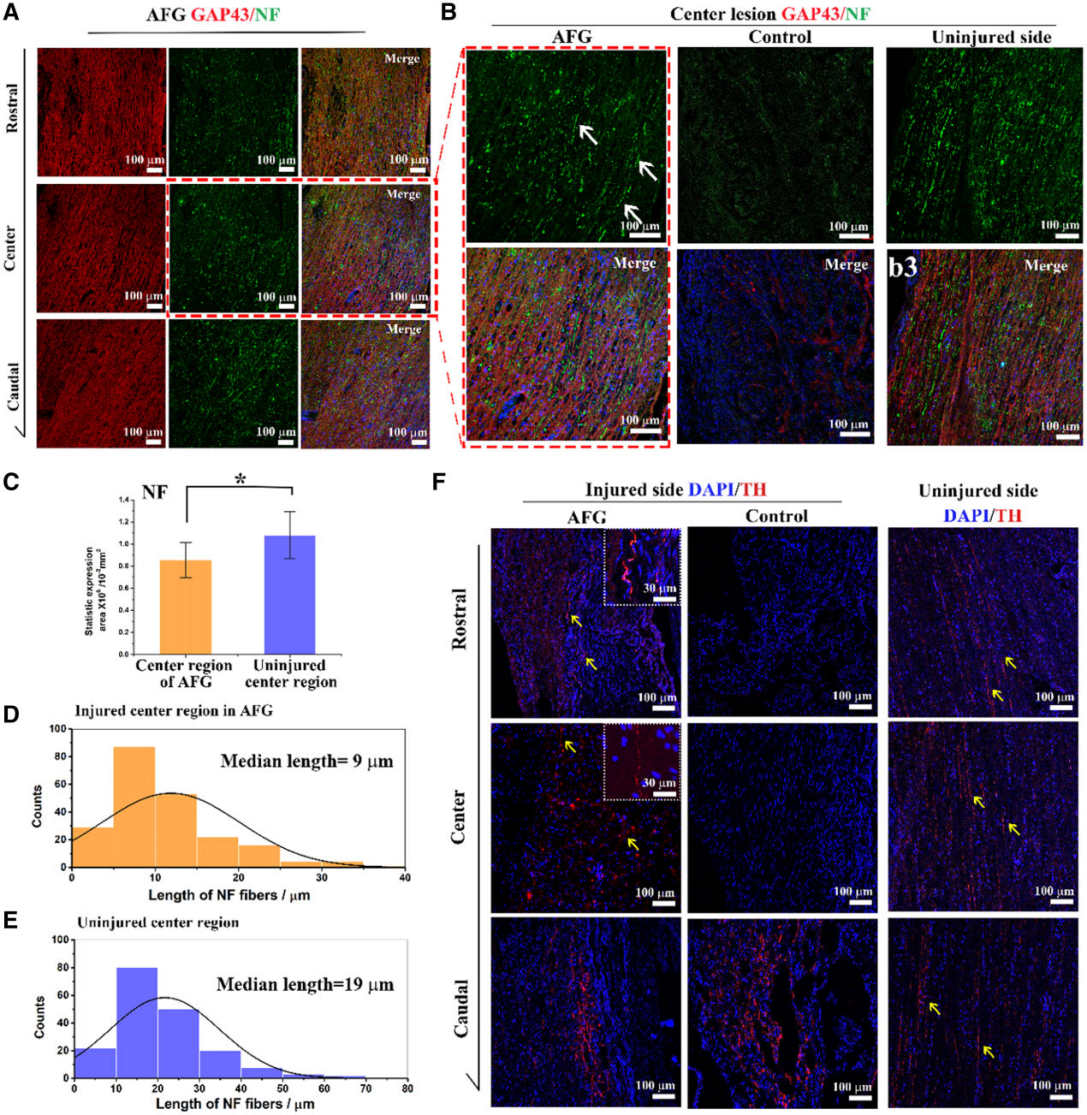
Immunofluorescence stain of axon in spinal cord. (**A**) Immunofluorescence stain of NF^+^ in green and GAP 43^+^ in red in rostral, center, and caudal portion of AFG spinal cord sections. (**B**) Expression of NF and GAP 43 merged images in the center region of AFG, control, and uninjured spinal cord. Arrows pointed out the obvious NF fiber. (**C**) NF expression area comparison between center regions of AFG and uninjured contralateral spinal cord. (**D** and **E**) Distribution of NF^+^ fiber length and calculated median length of AFG spinal cord and uninjured contralateral spinal cord. (**F**) Immunofluorescence stain of TH in the lesion of the AFG and control groups and uninjured spinal cord side. The yellow arrows highlighted the TH^+^ and squares in the top right corner showed the amplifying image of the TH^+^. (*P < 0.05).

To assess the regrowth of axons in the injured spinal cord, immunofluorescence staining of NF and GAP43 was used to identify the axons. As shown in Fig.  6, many NF^+^ fibers could be detected in the rostral, epicenter, and caudal regions crossing the lesion site of the injured spinal cord in the AFG group, which had co-expression of GAP43, indicating that AFG could assist the reconnection of the neuronal pathway via the sprouted neurites (Fig.  6A). Simultaneously, aligned long fibers co-expressed GAP43 and NF were also observed in the center of the lesion, suggesting deep migration of the sprouting axons. In contrast, only sporadic NF and GAP43 were observed in the control group, demonstrating the limited capacity of axon sprouting (Fig.  6B). Meanwhile, the contralateral spinal cord in the AFG group showed profuse and widely distributed NF^+^ and GAP 43^+^ (Fig.  6B and Supplementary Fig. S6), which indicated that the axons in the contralateral spinal cord were well retained. The NF^+^ expression level of the lesion center in the AFG group was similar (Fig.  6C) to that in the uninjured contralateral region, but the length of NF^+^ fiber was lower (AFG vs. uninjured contralateral, 9 μm vs. 19 μm, Fig.  6D and E).

Tyrosine hydroxylase (TH) plays an important physiological role in dopaminergic neurons and adrenergic neurons and often serves as a marker to visualize functional neurons [29]. The filamentous TH^+^ fibers were clearly visualized in the injured spinal cord of the AFG group throughout the defects from the rostral to center, to caudal regions (Fig.  6F, AFG), implying deep invasion and connection of functional neurons across the lesion. In the control group, numerous TH^+^ fibers were mainly found in the caudal region, few were noted in the rostral and center portions (Fig.  6F, control). Moreover, in the uninjured contralateral spinal cord of AFG, TH^+^ exhibited filamentous and uniform distribution and could be traced directionally between the rostral and caudal lesions (Fig.  6F, uninjured side). In aggregate, the regrowth of functional axons in the injured spinal cord can be improved by the implanted AFG, which might lead to the recovery of neuronal signal conduction.

## Discussion

The application of tissue engineering strategies for spinal cord regeneration and functional reconstruction has been extensively studied in the past decades. Although stem cells or bioactive agents are thought to be indispensable, optimizing bio-scaffolds by adjusting their biophysical and biochemical features for activating tissue regenerative potentials from a biomaterial perspective is important for clinical translation. The AFG has exhibited great potential to bridge the injured nerve tissue and guide endogenous axonal sprouting in rodents. Therefore, it is necessary to clarify whether it is feasible to ameliorate functional outcome by inducing white matter regeneration in a large animal SCI model with severe defects of the white matter and gray matter.

Our previous study showed a positive effect of the AFG in treating canines with SCI at the L2 level. The L2 segment in the lumbar spine is close to the cauda equina nerve, which is mainly composed of white matter nerve fibers with less gray matter. White matter regeneration and its effect on locomotor recovery, following SCI in the presence of gray matter damage, should be clarified to understand the biofunction of pure biomaterials. In this study, we evaluated the critical role of AFG in promoting white matter regeneration at a higher level (i.e. T10–12), which is located in the middle of the entire spinal cord with abundant ascending and descending tracts and comparable amounts of white matter and gray matter. In the present study, a high-resolution multi-shot DTI sequence was developed to evaluate the spatiotemporal alteration of the nerve fibers in white matter during the entire observation period (Fig.  3), which demonstrated the positive effect of AFG on inducing white matter regeneration. The DTI tractography results were highly consistent with the histological findings and locomotor outcomes. Obvious white matter regeneration composed of a large area of consecutive, compact, and aligned nerve fibers with positive expression of NF, GAFP, GAP43 and TH neural markers in the AFG group was widely observed, which contributed to the outstanding behavioral improvements. Generally, it could be concluded that AFG provided a functional bridge to facilitate axonal sprouting and white matter regeneration at the tissue level, which contributed to functional reconstruction during SCI recovery.

SCI is a complex pathological condition with destruction of both gray matter and white matter. Tissue loss after severe SCI results in cavity formation (i.e. syringomyelia) at the lesion site, which increases the barrier for structural rebuilding and functional restoration. Notably, the regenerative potentials for gray matter and white matter are considerably different due to the distinct cell densities; that is, neuron-rich gray matter is more difficult to rebuild without cell intervention [30, 31]. However, Davies [32] reported that degenerating white matter in injured spinal cord had intrinsic ability to regenerate, indicating the possibility of functional recovery after SCI by facilitating white matter regeneration. Giving the directional structural feature of spinal cord nerve fibers, it is noted that the graft with analogous nanoscale topography have better capacity to serve as an artificial ECM and restore the integrity of disconnected spinal cord [33, 34]. Pettigrew demonstrated that white matter only supported axonal growth in parallel direction and inhibited non-parallel growth, which is different from multidirectional axonal growth in gray matter [35]. Apparently, the axons in the white matter prefer to extend along the tract, emphasizing the critical role of the aligned geometry for axonal extension. During the past decades, a variety of bio-scaffolds have been developed to improve spinal cord regeneration. Aligned poly (ε-caprolactone-co-ethyl ethylene phosphate) and collagen composite scaffold could induce neurite growth [18, 36]. The linear-ordered BDNF binded collagen scaffold was effective in motor function recovery after SCI [22, 37]. Templated agarose scaffolds with linear arrays seeded with BDNF supported motor axon regeneration into a severe SCI model and organized axons into fascicles of highly linear configuration [38]. Unlike the abovementioned oriented scaffolds with growth factor seeded, AFG was designed to biomimic the native nerve and showed a hierarchically aligned nanofiber structure, thus had topographic advantage to facilitate white matter regrowth. Additionally, The moduli of elasticity of AFG 3.08 ± 0.28, which was close to that of the white matter in spinal cord: 3.4 ± 0.9 kPa in the axial section, 3.5 ± 0.5 kPa in the frontal section, and 2.8 ± 0.4 kPa in the sagittal section, respectively [39]. Therefore, the appropriate elastic modulus of AFG could be a positive cue for neurogenesis. Moreover, fibrin was the main component of the AFG. Due to the natural, biocompatible, and biodegradable properties, fibrin has been widely recognized as a promising biomaterial for tissue engineering in the spinal cord repairment [40, 41]. It is noteworthy that fibrin can interact with macrophages which play a crucial role in axon regeneration and remodeling after SCI. Hsieh reported that fibrin gel could prevent macrophages transforming to pro-inflammatory phenotypes when stimulated with lipopolysaccharide and interferon-γ, which were known to promote inflammation [41]. Similarly, Tanaka. showed fibrin hydrogel had a strong recruitment effect on anti-inflammatory macrophages [42]. The immunoregulatory effects of fibrin allowed AFG/fSAP reduced the pro-inflammatory macrophages accumulating at the lesion site and induced the infiltration of anti-inflammatory subtype. Also, AFG could be fully degradated few weeks after the implantation so that the implant did not cause any exogenous obstacles for tissue regeneraiton. Those mechanical and biological features abovementioned significantly promoted neuronal elongation without any additional growth factors [20].

Although our study showed the advantage of AFG implantation for facilitating white matter repair and locomotor recovery in canines, further research pertaining to the therapeutic approach to spinal cord repair should be considered. First, the interspecific difference in the canines and humans may lead to unsatisfactory effects when translating the AFG into clinical practice. Further studies in non-human primates are an unmet need in clinical trials of AFG. Second, the function of the spinal cord relies on the nerve fibers in white matter to conduct signals and synapses in gray matter to integrate the signals. Thus, white matter regeneration may be insufficient for complete functional recovery. A multifunctional tissue-engineered approach is required to improve the effect of AFG in nerve tissue repair, for example, loading various bioactive molecules, stem cells, or cell-originated vesicles on AFG, to modulate the complicated pathological process after SCI. Besides, we did not set another control group which was implanted with random fibrin hydrogel due to the consideration of animal ethics. Thus, we cannot compare the effects of the distributions of nanofibers on white matter regeneration in large animals.

## Conclusion

In this study, the AFG was implanted to direct neurogenesis in canine hemisected T12 spinal cord injuries. It was confirmed that AFG could induce histologically obvious white matter regeneration and significant motor functional restoration after SCI. Meanwhile, high-resolution multi-shot DTI showed the spatiotemporal dynamics of nerve regeneration rapidly crossing the lesion with the assistance of AFG. Our results suggest that AFG is a promising therapeutic vehicle for locomotion recovery by inducing rapid white matter regeneration in SCI, which pointed out its promising prospect in the clinic practice.

## Supplementary data

Supplementary data are available at *REGBIO* online.

## Funding

This research was supported by the Chinese National Natural Science Foundation (31771056, 31771052) and National Key Research and Development Project (2018YFB0704304, 2020YFC1107600).

*Conflicts of interest statement:* The authors declare that they have no competing interests.

## Supplementary Material

rbab069_Supplementary_Data
