# Supplementary material for: White matter regeneration induced by aligned fibrin nanofiber hydrogel contributes to motor functional recovery in canine T12 spinal cord injury
Source: Regen Biomater. 2021 Nov 29;9:rbab069. doi: 10.1093/rb/rbab069 (PMC9089163; doi:10.1093/rb/rbab069)
Supplement: rbab069_Supplementary_Data [file rbab069_supplementary_data.zip › Supporting information-RB.docx]

**Supplementary Information**

**White Matter Regeneration Induced by Aligned Fibrin Nanofiber Hydrogel Contributes to Motor Functional Recovery in Canine T12 Spinal Cord Injury**

Zheng Cao ^a,1^, Weitao Man ^a, c, 1^, Yuhui Xiong ^b,1^, Yi Guo ^c^, Shuhui Yang ^a^, Dongkang Liu ^c^, He Zhao ^a, d^, Yongdong Yang ^d^, Shenglian Yao ^e^, Chuzhong Li ^f^, Lingyun Zhao ^a^, Xiaodan Sun ^a^, Hua Guo ^b, *^, Guihuai Wang ^c, *^, Xiumei Wang ^a, *^

^a^ State Key Laboratory of New Ceramics and Fine Processing, Key Laboratory of Advanced Materials, School of Materials Science and Engineering, Tsinghua University, Beijing 100084, China

^b^ Center for Biomedical Imaging Research, Tsinghua University, Beijing 100084, China

^c^ Department of Neurosurgery, Beijing Tsinghua Changgung Hospital, School of Clinical Medicine, Tsinghua University, Beijing,102218, China

^d^ Department of Orthopedics, Dongzhimen Hospital, Beijing 100007, China

^e^ School of Materials Science and Engineering, University of Science and Technology Beijing, Beijing 100083, China

^f^ Beijng Neurosurgical Institute, Beijing Tiantan Hospital, Beijing 100070, China

^1^ These authors contributed equally to this work.

^*^ Correspondence address. State Key Laboratory of New Ceramics and Fine Processing, Key Laboratory of Advanced Materials, School of Materials Science and Engineering, Tsinghua University, Beijing 100084, China. Tel: +86-010- 62782966; E-mail: wxm@mail.tsinghua.edu.cn (X.W.), youngneurosurgeon@163.com (G.W.), hua.guo@gmail.com (H.G.)


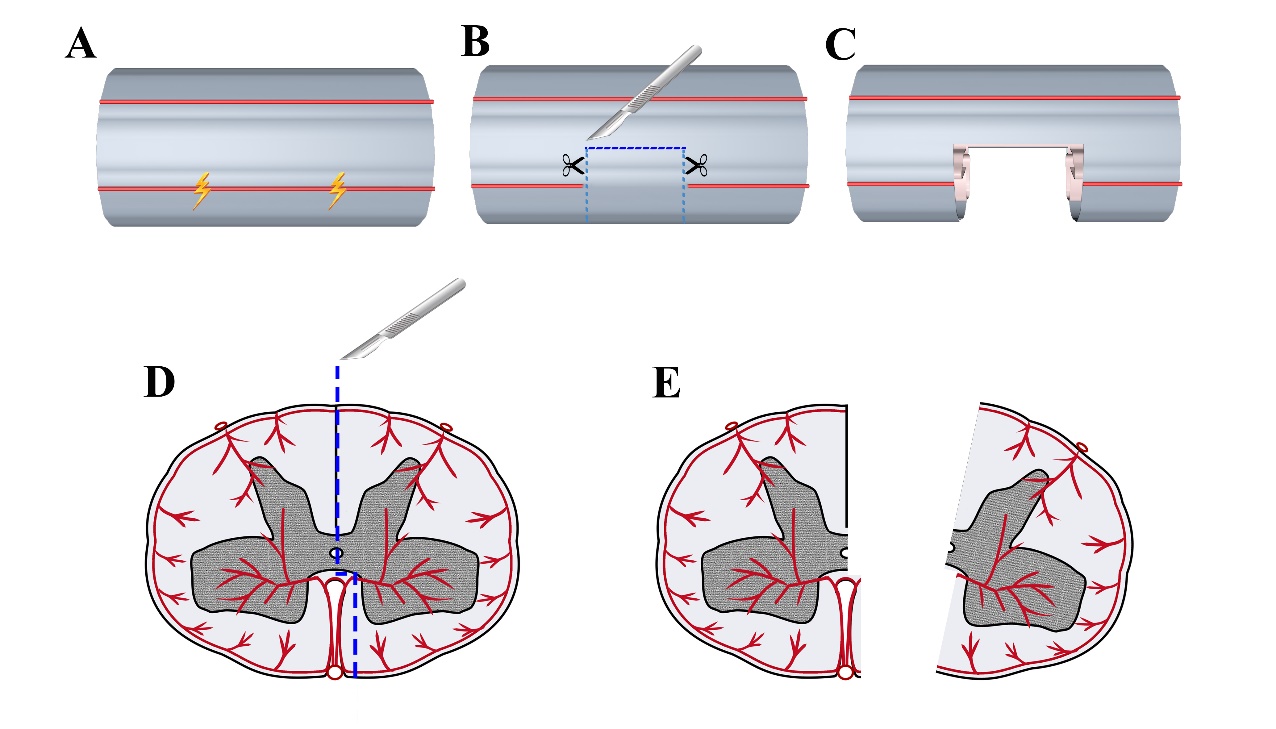


Figure S1 Surgery process of the hemisection SCI. (A) Coagulation the dorsal spinal vessels. (B) Manipulation the defects at the posterior median fissure. (C) Hemisection lesion. (D) Transection view of (B). (E) Transection view of (C).


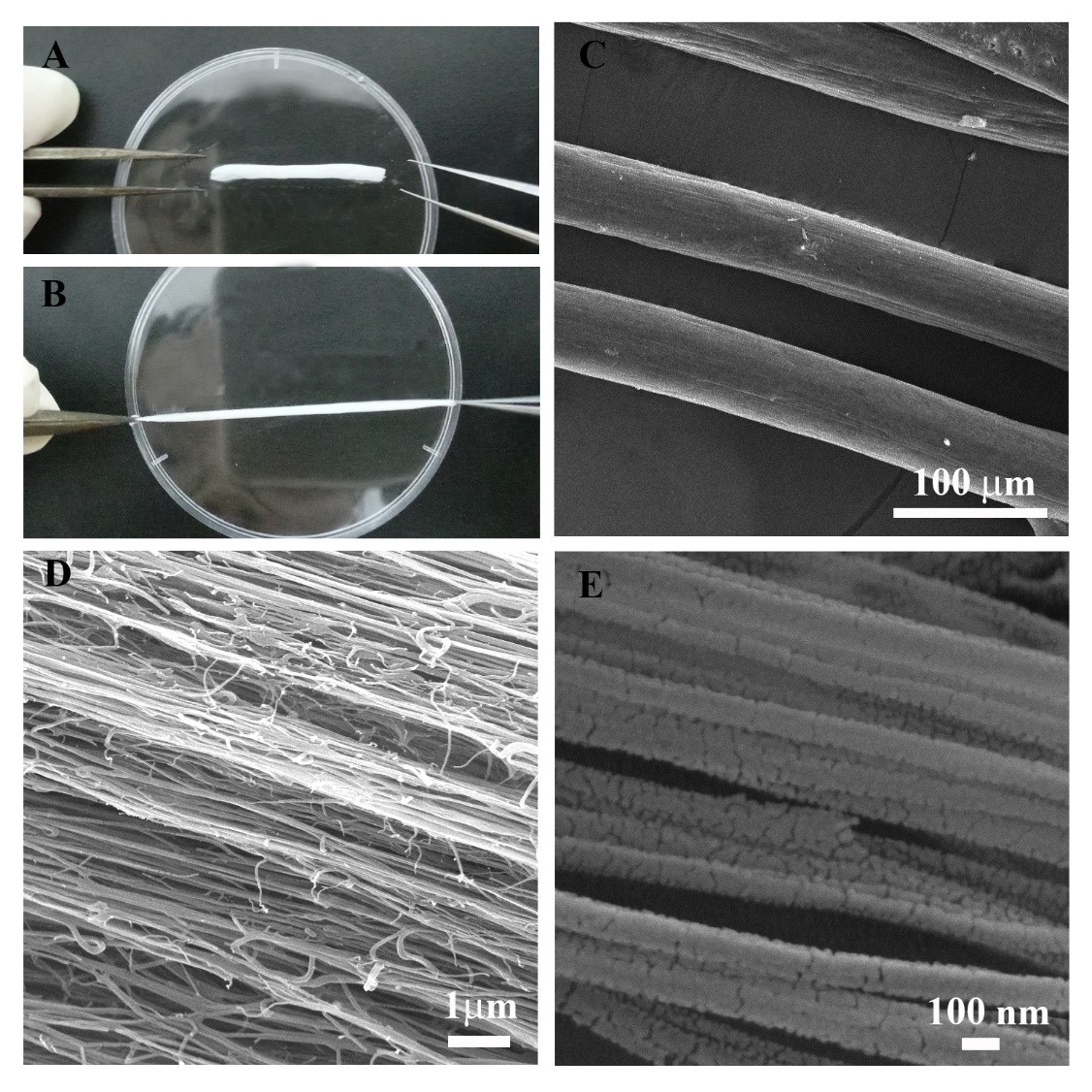


Figure S2 Characterization of AFG. (A) Macroscopic view of AFG. (B) The stretched AFG. (C-E) The SEM images of the AFG.


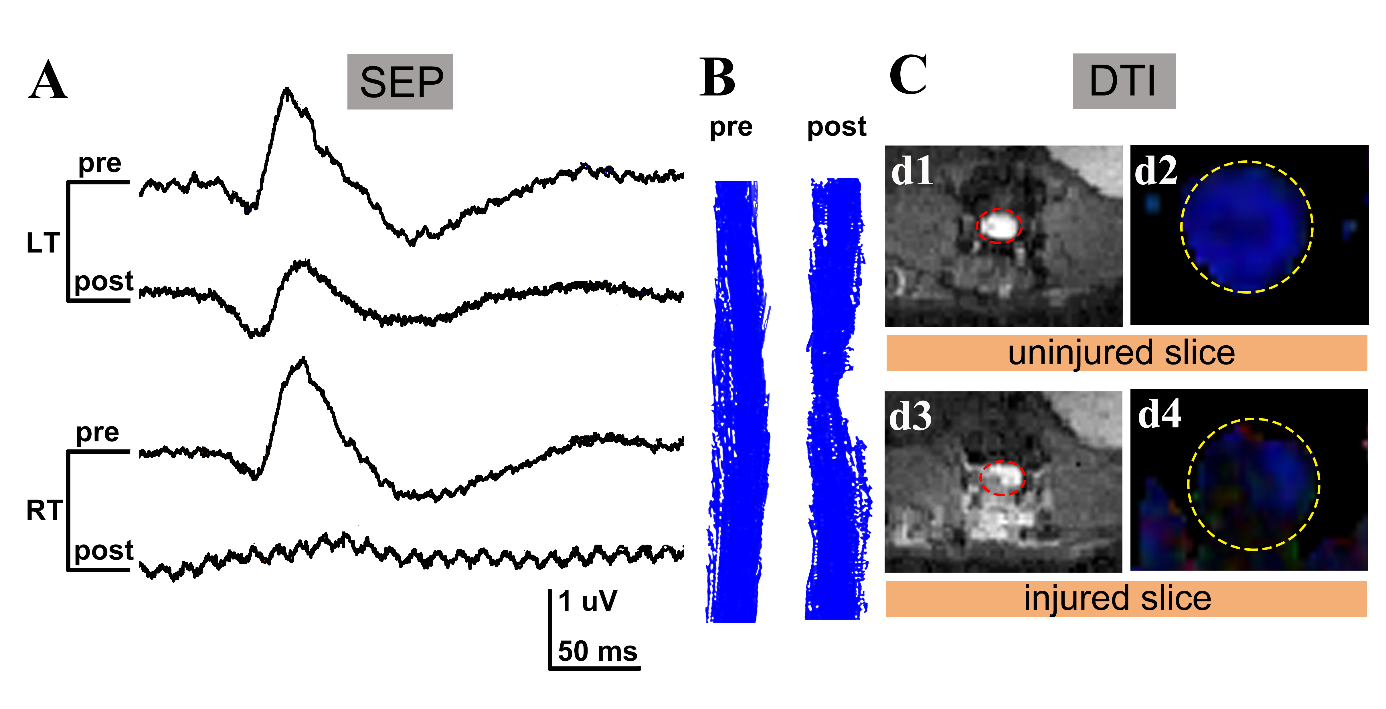


Figure S3 (A) Monitored SEP test during surgery, which respectively showed SEP signal pre- and postoperatively in the left (LT) and right (RT) legs. (B) DTI images in coronal and (C) cross section views showed the hemisected SCI injury.


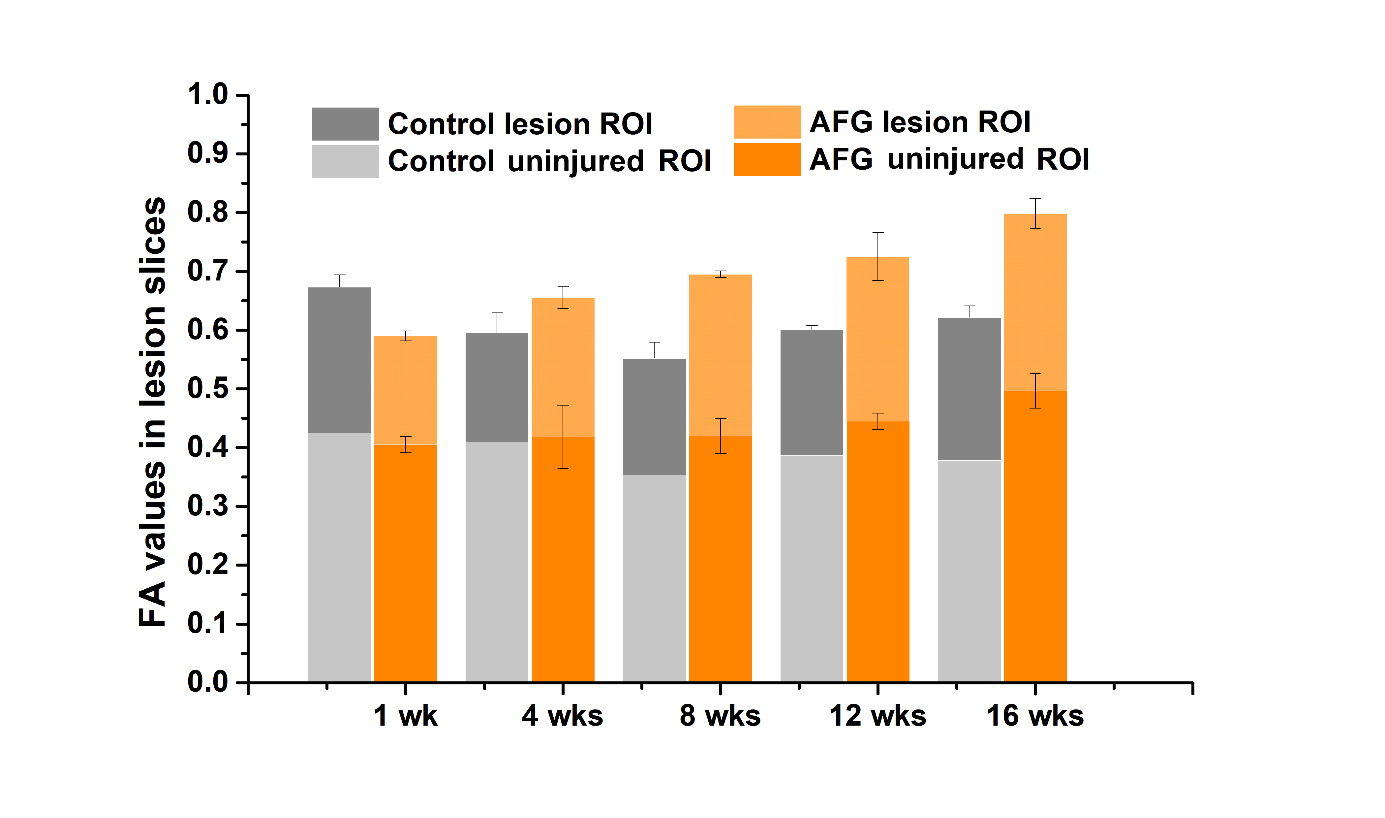


Figure S4 Accumulated FA values attributed to the lesion and uninjured regions in AFG and control group from 1 wk to 16 wks.


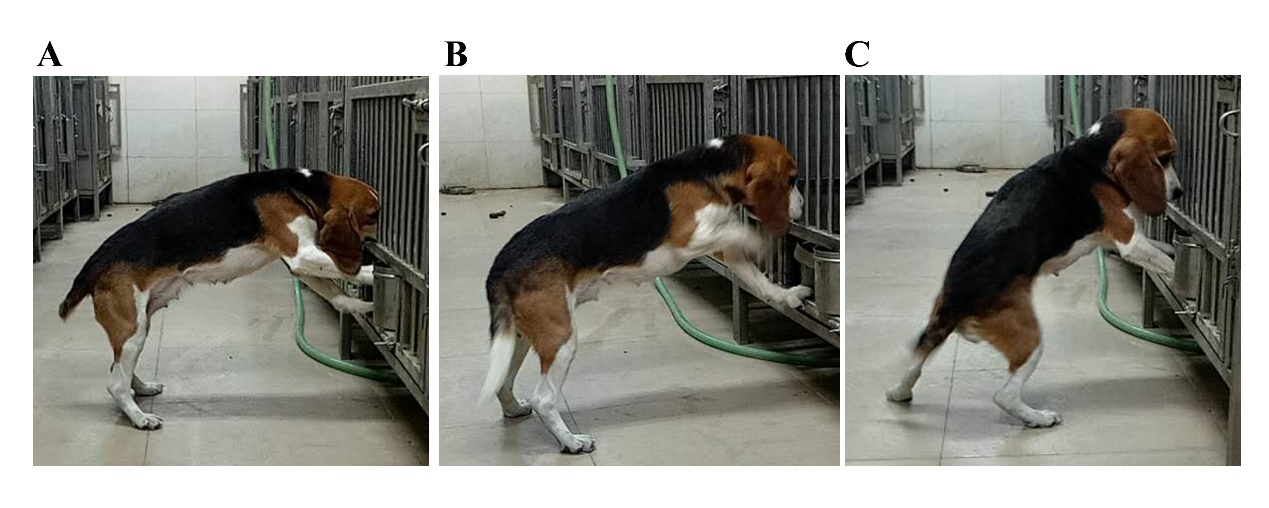


Figure S5 Pictures of the dog with hind limbs supportting the whole body weight. From A to C the center of the gravity gradually rearward moved to the hind limbs.


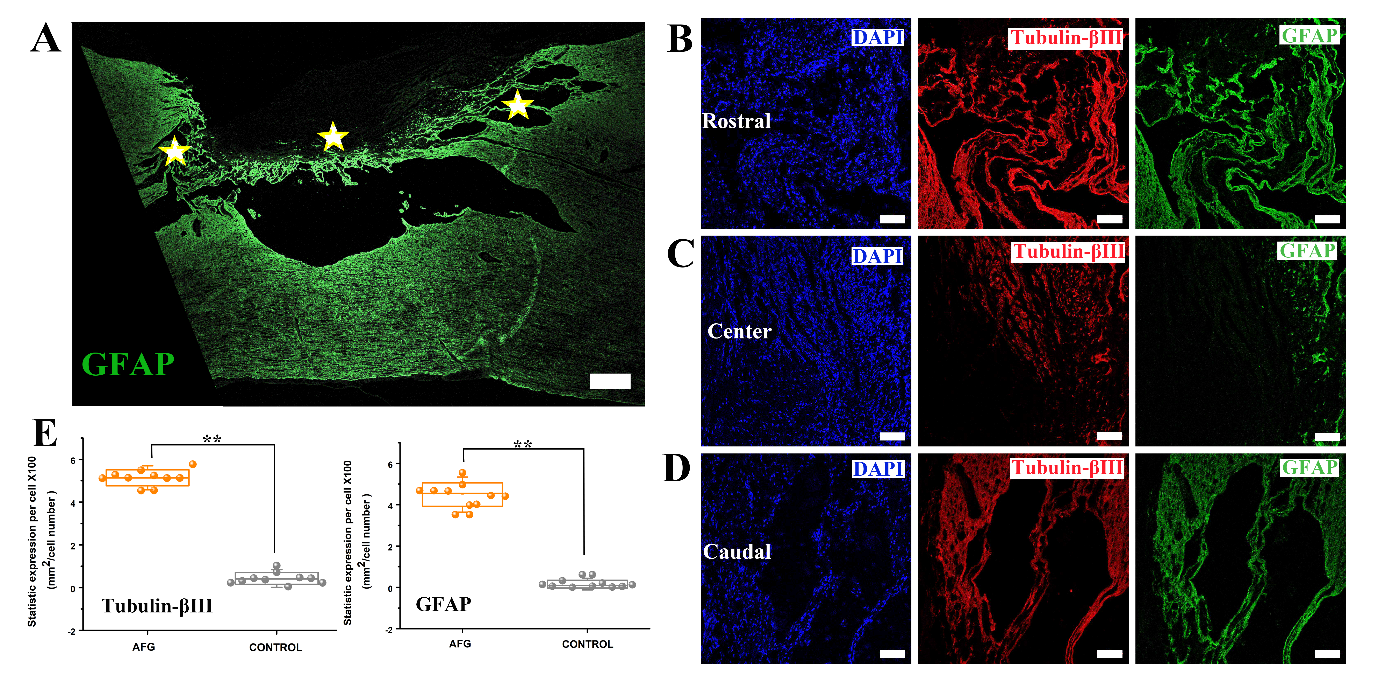


Figure S6 (A) Full immunofluorescence view of the spinal cord in control group (white bar is 500 μm) marked with GFAP, the yellow stars refer to the rostral, center and caudal lesion (white bar is 500 μm). (B-D) Tubulin-βⅢ^+^ I and GFAP^+^ images of control spinal cord in rostral, center and caudal regions respectively (white bar is 100 μm). (E) expression activity (ratio of fluorescence intensity to cell counts) of Tubulin-βⅢand GFAP compared between AFG and control in center lesion. (**P<0.01)


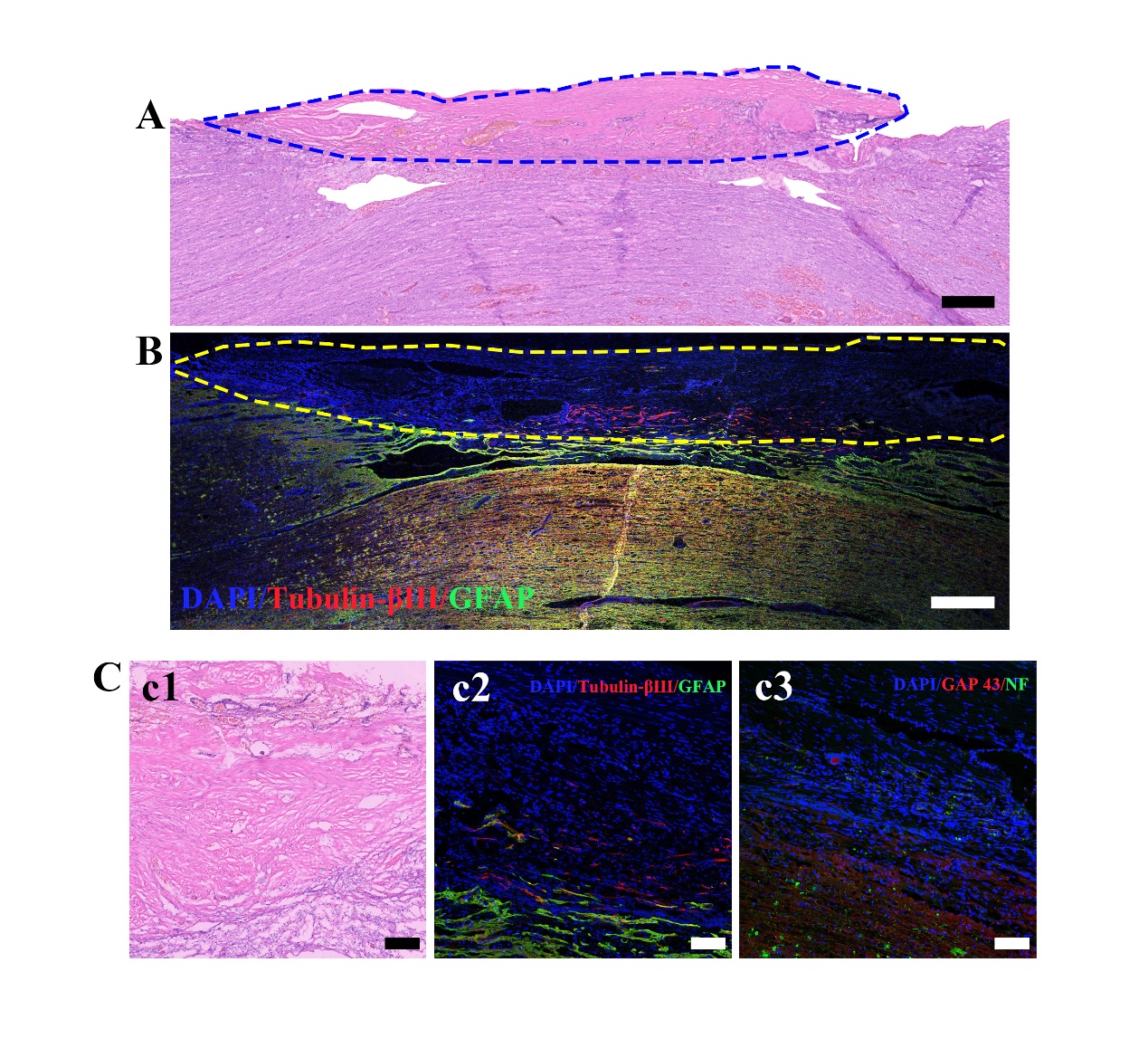


Figure S7 HE and immunofluorescence of the fringe tissue of AFG spinal cord sections. (A) Full view of the HE stain of the fringe tissue in blue circle (black bar is 500 μm). (B) Immunofluorescence of Tubulin-βⅢ, GFAP and DAPI of the tissue at the edge in the yellow circle (white bar is 400 μm). (C) Partial HE stain (c1, black bar is 200 μm), Tubulin-βⅢ, GFAP and DAPI (c2, white bar is 100 μm) and GAP43, NF and DAPI immunofluorescence stains (c3, white bar is 100 μm).


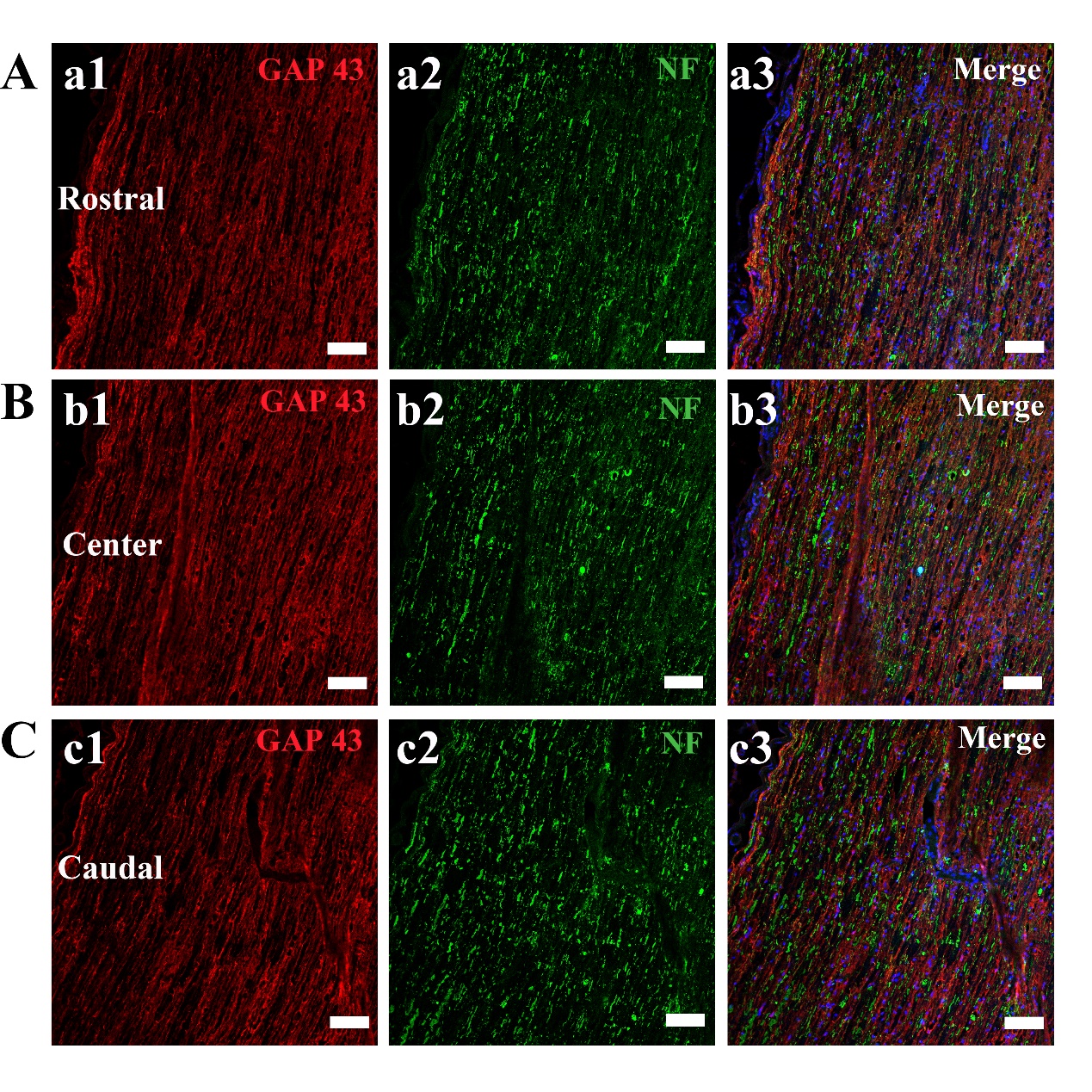


Figure S8 Immunofluorescence stain of axon in uninjured contralateral spinal cord. (A-C) Spinal cord stained with NF and GAP 43 in rostral, center and caudal portion respectively, in which a1-c1 referred to GAP 43+ in red color, a2-c2 were NF+ stained in green and a3-c3 were merged images (white bar is 100 μm).

Video S1 Surgery process of the hemisection SCI.

Video S2 Locomotor behavior videos of AFG dogs at 1 wk, 4 wks, 8 wks and 16 wks after the surgery.

Video S3 Locomotor behavior videos of control dogs at 1 wk, 4 wks, 8 wks and 16 wks after the surgery.

Video S4 Moments of the AFG and control dogs climb the steps.
